# Within-Subject Reliability and between-Subject Variability of Oxidative Stress Markers in Saliva of Healthy Subjects: A Longitudinal Pilot Study

**DOI:** 10.1155/2017/2697464

**Published:** 2017-11-15

**Authors:** Iva Z. Alajbeg, Ivana Lapić, Dunja Rogić, Lea Vuletić, Ana Andabak Rogulj, Davor Illeš, Dubravka Knezović Zlatarić, Tomislav Badel, Ema Vrbanović, Ivan Alajbeg

**Affiliations:** ^1^Department of Removable Prosthodontics, School of Dental Medicine, University of Zagreb, Zagreb, Croatia; ^2^Department of Laboratory Diagnostics, University Hospital Centre Zagreb, Zagreb, Croatia; ^3^Department of Physiology, School of Dental Medicine, University of Zagreb, Zagreb, Croatia; ^4^Department of Oral Medicine, School of Dental Medicine, University of Zagreb, Zagreb, Croatia; ^5^Department of Dentistry, University Hospital Centre Zagreb, Zagreb, Croatia

## Abstract

The present study evaluated diurnal variations and day-to-day fluctuations of salivary oxidative stress (OS) markers in healthy adult individuals. Whole unstimulated saliva was collected at 2 time intervals over 3 consecutive days. Glutathione peroxidase (GPX), superoxide dismutase (SOD), total antioxidant capacity (TAC), and uric acid (UA) were analyzed using spectrophotometric methods, while 8-hydroxydeoxyguanosine (8-OHdG) and malondialdehyde (MDA) were determined using immunoassays. No significant differences for salivary OS markers between men and women were observed. For all examined OS markers, no significant day-to-day variations were demonstrated. Significant diurnal variations were found in salivary GPX, TAC and MDA levels. For SOD, TAC, GPX, and UA, good-to-moderate intraindividual coefficients of variations (CVs) were observed in more than 75% of the subjects. For MDA and 8-OHdG, intraindividual CVs > 35% were observed in 60% and 40% of the subjects, respectively. Between-subject variance was wide for all examined OS markers (CV% 30.08%–85.70%). Due to high intraindividual variability in the salivary concentrations of MDA and 8-OHdG, those markers cannot be reliably verified based on single measurements and multiple measurements over several days would provide more reliable information. Salivary SOD, TAC, GPX, and UA proved stable across three days of measurement. *Trial Registration*. ClinicalTrials.gov NCT03029494. Registered on 2017-01-19.

## 1. Introduction

It has been suggested that oxidative stress is a result of the imbalance of oxidant/antioxidant status in biological systems, due to either an excessive production of free radicals or a reduction in the effectiveness of the antioxidant system. Although recent evidence has shown that a certain degree of oxidative stress is a necessary component of intracellular signaling [1], excessive levels of oxidative stress can cause serious damage to biomolecules [2].

Antioxidant defense systems can be divided into two categories: enzymatic and nonenzymatic. The nonenzymatic antioxidants include vitamins C and E, uric acid (UA), and albumin, while the major enzymatic antioxidants are glutathione peroxidase (GPX), superoxide dismutase (SOD), and catalase. Although the concentrations of individual antioxidants can be determined separately, the more conventional approach is to measure total antioxidant capacity (TAC), the cumulative effect of all antioxidants present in biological samples [3].

A number of biomarkers are routinely used as a measure of oxidative stress, including malondialdehyde (MDA), a product of lipid peroxidation [4, 5], and 8-hydroxydeoxyguanosine (8-OHdG), a marker of deoxyribonucleic acid oxidative damage [6, 7].

It is believed that oxidative stress plays a significant role in the development of many diseases [8–11]. Various sources are used for the assaying of disease biomarkers, including synovial fluid, tissue samples, blood, and urine [12–15]. Because oxidative stress is thought to be involved in the pathogenesis of numerous oral diseases, such as lichen planus [16], oral cancer [17], periodontitis [18, 19], and temporomandibular disorders [20], saliva is a highly desirable source for assaying potential biomarkers for those diseases. Saliva is also used to assess the possibility of therapeutic outcomes for these diseases. Rodríguez de Sotillo et al. [21] compared salivary and serum levels of oxidative stress biomarkers between patients experiencing pain caused by temporomandibular muscle and joint disorders and healthy subjects. Their results demonstrated significant association between pain severity and salivary biomarkers indicative of oxidative stress, suggesting their potential diagnostic and therapeutic importance. Oxidative stress biomarkers have been evaluated in patients with various other oral diseases to provide a basis for their early assessment as well as to explain the potential mechanisms involved in chronic pain [22, 23].

Regardless of this finding, there is little data available that measures the level of oxidative stress markers in human saliva in a healthy population. Most studies have used a case-control design, comparing patients with healthy controls, and little information regarding standardization and validation of method can be found. In other words, the results of those studies could be due to possible random variation in measurements.

A recent study by Kamodyová et al. [24], conducted on healthy individuals, observed significant diurnal variations in the level of advanced glycation end products (AGEs) and ferric reducing antioxidant power (FRAP). Other salivary biomarkers of oxidative stress and antioxidant status, including thiobarbituric acid reactive substances (TBARS) and TAC, did not differ significantly during the day. Their study also revealed a significant influence of external factors, vitamin C administration and tooth brushing, on the levels of measured biomarkers. However, this study did not evaluate day-to-day fluctuations of oxidative stress markers over a short period of time. To the best of our knowledge, the only attempt to assess intraindividual and interindividual day-to-day variability of salivary oxidative stress markers in young healthy subjects was made by Lettrichová et al. [25]. The authors reported high intra- and interindividual variability of all the markers measured (AGEs, advanced oxidation protein products (AOPP), TAC, and FRAP) suggesting that such results make interpretation of individual values difficult. Behuliak et al. [26] also analyzed the intra- and interindividual variability of salivary oxidative stress markers in young healthy individuals, but only for TBARS (TBARS represent a heterogeneous group of compounds majority of which are malondialdehydes).

The purpose of our pilot study is to evaluate the diurnal variability of salivary GPX, SOD, TAC, UA, 8-OHdG, and MDA in healthy adult individuals over three consecutive days. An understanding of the interindividual, intraindividual diurnal, and day-to-day variability of oxidative stress markers will allow further experiments to be planned. If high repeatability is observed, our results will serve as a standardized reference, to which future comparisons can be made. Furthermore, if high repeatability is established, we will not need to perform a series of measurements within each subject.

Our central hypothesis is that we will not observe intraindividual differences if sampling is performed at different times of the day or repeatedly on different days. We also hypothesize that the tested parameters will not vary substantially between healthy individuals.

## 2. Methods

### 2.1. Ethics, Consent, and Permissions

The study was approved by the Ethics Committee of the School of Dental Medicine, University of Zagreb (01-PA-26-6/15, item 3.2.). Written informed consent was obtained from all subjects after a detailed explanation of the study. All experimental procedures were conducted in accordance with the ethical standards of the Helsinki Declaration. Recruitment and sample collection were performed between April 2016 and October 2016.

### 2.2. Subjects

Power analysis, performed to estimate sample size, was based on Kato et al.'s blood biomarkers variability [27], with a within-group variance of 0.84. The effect size was hypothesized to be 0.06. Accordingly, with alpha = 0.05 and power = 0.80, the projected sample size needed with this variance (GPower 3.1) was *N* = 15 for the within-group comparison, and 6 measurements per individual would be necessary in order to obtain reliable data.

Fifteen healthy adult volunteers, nine females and six males (mean age 38.73 ± 5.18 years), were recruited for this study. Their body mass index ranged from 18.5 to 23.5 kg/m^2^. All subjects had normal systolic and diastolic blood pressures, and their resting heart rates were in the lower normal limit for healthy subjects.

All subjects were nontobacco users who had abstained from any dietary aids that might affect the outcome results. They did not take medications or supplements that could influence oxidative stress measures nor did they have any metabolic, heart, or muscle abnormalities. In addition, all maintained good oral hygiene and had good oral health with no apparent dental cavities, gingival, and periodontal diseases or mucosal lesions. All had their natural teeth.

### 2.3. Specimen Collection

Whole unstimulated saliva samples were collected at 2 time intervals, 7 AM and 5 PM, on 3 consecutive days. In total, 90 samples from 15 subjects were collected and analyzed.

Subjects were instructed to fast before morning saliva collection, and not to eat, drink anything except water, or use chewing gum for 2 hours before afternoon saliva sampling. Tooth brushing was also forbidden prior to collection in order to avoid contamination of saliva samples with blood. All saliva samples were collected after rinsing the mouth for 30 seconds with clean water. During collection, subjects were asked not to talk or think about food and to attempt not to generate saliva.

Five milliliters of whole unstimulated saliva was collected into a graduated tube (50 mL, self-standing centrifuge tubes, Ratiolab, Germany). The time needed to collect the sample was used to calculate salivary flow rate (mL/min). Mean flow rate was 0.41 mL/min (range 0.11–0.96 mL/min) for morning and 0.53 mL/min (range 0.12–0.77 mL/min) for afternoon collection.

Saliva aliquots (1 mL) were stored at −80°C until analyzed. Saliva samples were thawed and centrifuged prior to analysis (1000 ×g, 5 min).

### 2.4. Biochemical Sample Analysis

MDA levels were measured using an MDA adduct competitive enzyme-linked immunosorbent assay (ELISA) kit (Kamiya Biomedical Company, Seattle, WA, USA). The test involves the addition of unknown samples to an MDA conjugate precoated ELISA plate, followed by the addition of an anti-MDA polyclonal antibody and a horseradish peroxidase (HRP) conjugate secondary antibody. The content of MDA protein adducts in saliva samples was determined by measuring the absorbance of an enzyme conversion product after the addition of an enzyme substrate at a specified wavelength (450 nm). MDA adduct level reflects the quantity of MDA that combines with proteins in the process of lipid peroxidation. The range of the assay is 6–1500 pm/mL; thus, the minimum detectable concentration of MDA adducts less than the lowest standard is reported as <6 pm/mL The amount of MDA adduct in the used standards was predetermined by the manufacturer of a TBARS assay kit; results obtained with this kit can be used to compare findings with those from other studies analyzing MDA. Within-laboratory CV for the MDA adduct assay was determined by 10 replicate measurements of one saliva sample, and the CV (%) obtained was 16.6% at 134 pmol/mL concentration.

8-OHdG levels were measured in a similar manner, using a highly sensitive ELISA competitive kit (Japan Institute for the Control of Aging, Shizuoka, Japan). This kit uses an 8-OHdG monoclonal antibody to bind, in a competitive manner, 8-OHdG in the analyzed samples or 8-OHdG prebound to the wells of the microtiter plate. Immobilized 8-OHdG is detected with an HRP conjugate secondary antibody and tetramethylbenzidine as a chromogenic substrate, causing color development measured at 450 nm. Intra-assay variation was determined by measuring one saliva sample 10 times in a single batch, and the obtained CV (%) of the 8-OHdG assay was 13.9% at 1.19 ng/mL concentration.

SOD, GPX, and TAC were measured utilizing commercial colorimetric reagent kits RANSOD, RANSEL, and TAS (Randox Laboratories Ltd., Crumlin, United Kingdom), respectively, and applied on a Cobas c501 biochemistry autoanalyzer (Roche Diagnostics, Mannheim, Germany).

The RANSOD test measures SOD levels by employing xanthine and xanthine oxidase (XOD) to generate superoxide radicals, which subsequently react with 2-(4-iodophenyl)-3-(4-nitrophenol)-5-phenyltetrazolium chloride (INT), forming a red formazan dye. Because the role of SOD is to enhance the dismutation of superoxide radicals, its activity is easily measured by the degree of inhibition of this reaction. One unit of SOD corresponds to a 50% inhibition. The intra- and interassay variabilities for the RANSOD test were 4.6% and 7.1%, respectively, as declared by the manufacturer.

The RANSEL test measures GPX levels by determining the decrease in absorbance at 340 nm of NADPH, which is converted to NADP+ in the oxidation reaction of glutathione catalyzed by glutathione peroxidase. The intra- and interassay variabilities for the RANSEL test were 4.9% and 7.3%, respectively, as determined by the manufacturer.

A TAS kit measures the TAC of the samples in a reaction catalyzed by peroxidase, producing a radical cation, ABTS∗+, whose absorbance is consequently measured at 600 nm. Antioxidant capacity is determined by the capability of antioxidants present in the analyzed sample to suppress this reaction and subsequent color development. The intra-assay variability of this assay was 2.8% at 1.5 mmol/L TAC and was determined by repeated measurements of saliva samples.

Uric acid was measured by the enzymatic uricase method, utilizing commercially available Roche Diagnostics reagents applied to a Cobas c501 Biochemistry analyzer (Roche Diagnostics, Mannheim, Germany). Within-run and between-run coefficients of variation (CV%) of the uric acid assay were 1.4% and 1.8%, respectively, and were determined by repeated measurements of commercial control samples during method validation.

The amount of total proteins in saliva samples was determined by a commercially available Roche Diagnostics automated turbidimetric urinary and cerebrospinal fluid (CSF) protein assay whose measuring range (0.02–2 g/L) and lower detection limit (0.02 g/L) covered the expected values and had satisfactory sensitivity for saliva samples. Within-run and between-run CVs of the assay were 0.9% and 1.0%, respectively. Analysis was performed on a Cobas c501 biochemistry analyzer (Roche Diagnostics, Mannheim, Germany). All parameter values were normalized to total protein concentration.

All measurements were performed using one reagent kit for each parameter.

Laboratory measurements were performed at the Department of Laboratory Diagnostics, University Hospital Centre Zagreb.

### 2.5. Statistical Analysis

Data analyses were performed using SPSS 17.0 (Chicago, IL, USA) with alpha set at *p* < 0.05. The distributions of data were tested for normality using the Shapiro-Wilk test. Before performing statistical analysis, a log transformation was used for those biomarkers that were nonnormally distributed.

Repeated measure ANOVA was used, with the time of day (morning/afternoon) and day (1/2/3) as within-factors and gender as between-factor.

Within-subject reliability over 3 consecutive days was calculated at two time intervals (7 AM and 5 PM) using intraclass correlation coefficients (ICCs) (two-way mixed model effects) for salivary GPX, SOD, TAC, UA, 8-OHdG, and MDA. The single measure ICC was reported along with the associated 95% confidence interval. ICC values were interpreted as follows: ≥0.75 indicated excellent reproducibility, 0.35–0.75 indicated moderate reproducibility, and <0.35 indicated poor reproducibility. Between-day variance for each OS marker at both time intervals was expressed as coefficient of variation percentage (CV%) (between-day SD/between-day mean) × 100.

Group means and standard deviations for each biomarker at both time intervals over the 3 days were calculated. Between-subject variability was expressed as group coefficient of variation percentage (CV%) ((group SD/group mean) × 100). CV was regarded as very good if CV ≤ 10%, good if 10% < CV ≤ 25%, moderate if 25% < CV ≤ 35%, and poor if CV > 35%.

## 3. Results

Fifteen participants completed all three testing sessions. No significant difference in age between genders was found (*p* > 0.05). None of the participants withdrew from the study.

**Figure 1:**
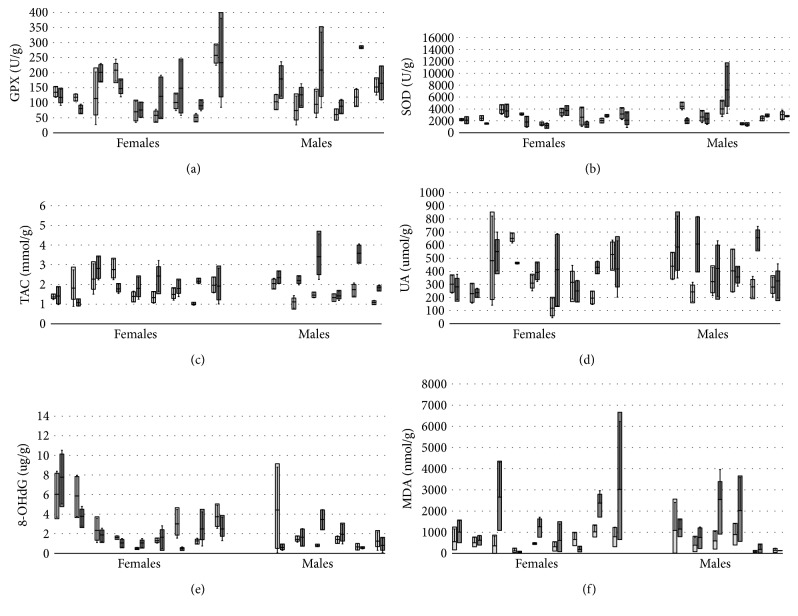
Salivary glutathione peroxidase (GPX) (a), superoxide dismutase (SOD) (b), total antioxidant capacity (TAC) (c), uric acid (UA) (d), 8-hydroxydeoxyguanosine (8-OHdG) (e), and malondialdehyde (MDA) (f) levels in female and male participants. Box plot represents individual subjects' data. Data are presented as upper value, lower value, mean, and standard deviation. Light grey represents morning measurements; dark grey represents afternoon measurement.

Mean salivary levels of GPX, SOD, TAC, UA, 8-OHdG, and MDA (Figures 1(a), 1(b), 1(c), 1(d), 1(e), and 1(f)) did not vary significantly between males and females (*p* > 0.05). Repeated measure ANOVA was used to evaluate diurnal variations and day-to-day fluctuations of oxidative stress marker levels. For all examined salivary oxidative stress markers, no significant day-to-day variations were found (*p* > 0.05). Significant diurnal variations were observed in salivary GPX levels (*F* = 9.440; *p* = 0.009) and TAC levels (*F* = 9.291; *p* = 0.009), with significantly lower values in the morning than in the afternoon, which suggests that diurnal rhythm is affected by these markers. Salivary UA concentrations were slightly higher in the afternoon, but the differences between morning and afternoon concentrations were not statistically significant. Significant diurnal variations were also observed in salivary MDA levels (*F* = 6.298; *p* = 0.026); concentrations were lower in the morning and significantly higher in the afternoon. The levels of salivary SOD and 8-OHdG did not differ significantly during the day (*p* > 0.05).

Since no gender differences were found, male and female data were combined for further analyses.

**Figure 2:**
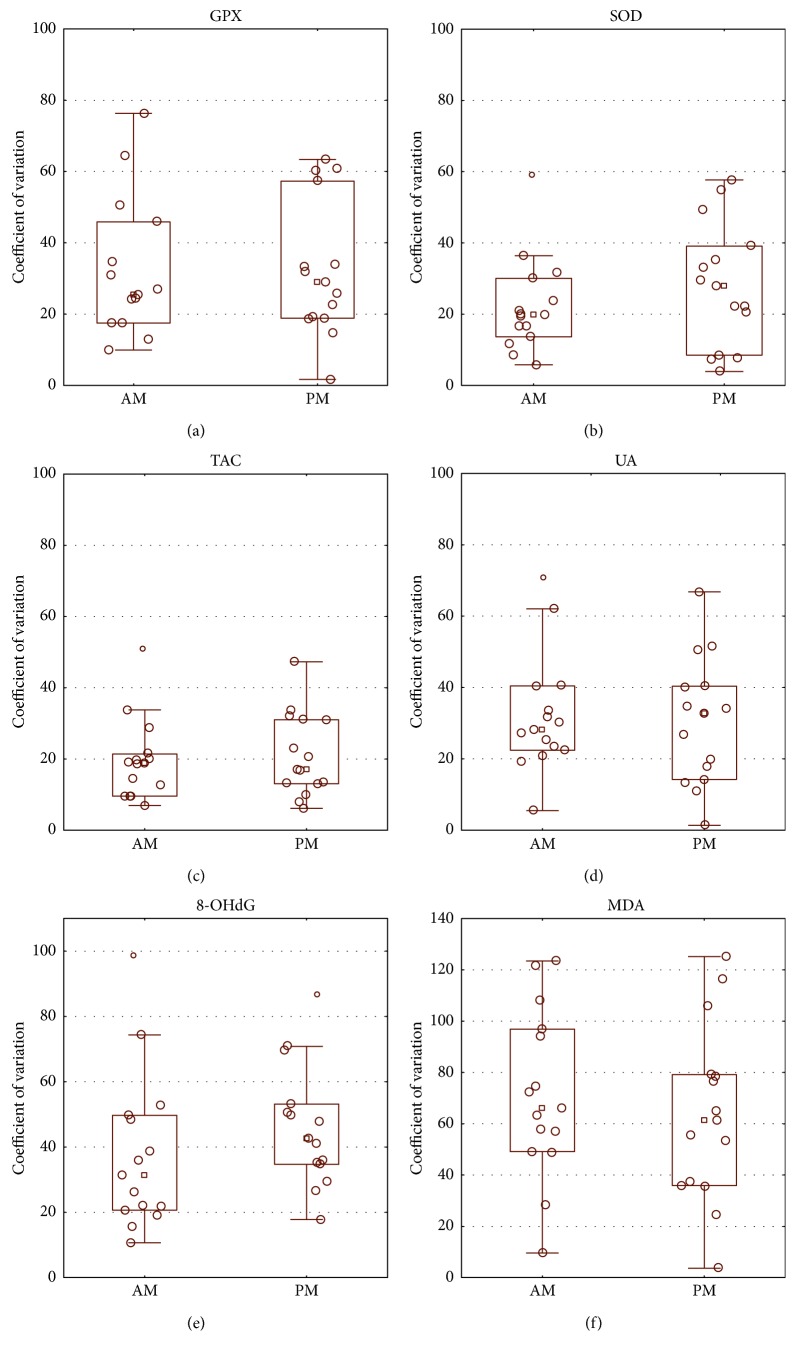
Intraindividual variability of glutathione peroxidase (GPX) (a), superoxide dismutase (SOD) (b), total antioxidant capacity (TAC) (c), uric acid (UA) (d), 8-hydroxydeoxyguanosine (8-OHdG) (e), and malondialdehyde (MDA) (f).

Intraindividual variability for all oxidative stress markers was calculated. For salivary GPX, interday CV < 35% was observed in 75% of the subjects (Figure 2(a)). Interday CV < 25% was observed in 75% of the subjects for salivary SOD (Figure 2(b)), and in more than 80% of the subjects for salivary TAC (Figure 2(c)). For salivary UA, in 75% of the subjects, interday CV < 35% was noted (Figure 2(d)). Intraindividual variability for 8-OHdG and MDA ranged widely. Interday CV > 35% for 8-OHdG was observed in 40% of the subjects (Figure 2(e)). For salivary MDA, interday CV > 35% was observed in 60% of the subjects (Figure 2(f)).

Within-subject reliability (Table 1) across the three test days was high for salivary SOD and 8-OHdG both for morning (ICC for SOD *r* = 0.76; ICC for 8-OHdG *r* = 0.82) and for afternoon (ICC for SOD *r* = 0.77; ICC for 8-OHdG *r* = 0.83) measurements. Between-day variance across the three test days ranged from 4.22% to 7.6% for SOD and from 6.68% to 14.5% for 8-OHdG. Salivary GPX showed greater reliability while tested in the morning (ICC *r* = 0.86) compared to the afternoon (ICC *r* = 0.61), although between-day variance was low at both times of the day (morning CV% = 3.28; afternoon CV% = 4.40%). Salivary TAC showed greater reliability while tested in the afternoon (ICC *r* = 0.81) compared to that in the morning (ICC *r* = 0.76), with between-day variance of 4.06% for morning and 6.83% for afternoon measurements. Lower reproducibility for afternoon salivary UA (ICC *r* = 0.51) compared to that for morning was observed (ICC *r* = 0.72), with between-day CV% less than 10%. Salivary MDA showed higher reliability when tested in the afternoon (ICC *r* = 0.86) compared with morning measurements (ICC *r* = 0.34).

Between-subject variability was the highest for 8-OHdG and MDA, for both morning (8-OHdG CV% = 75%; MDA CV% = 59.13%) and afternoon measurements (8-OHdG CV% = 85.70%; MDA CV% = 82.65%). Other oxidative stress markers also showed wide between-subject variability, with CV% between 30.08% and 49.66% for morning and 30.28% and 57.24% for afternoon measurements (Table 2).

## 4. Discussion

Numerous studies have examined the relationship between oxidative stress and pathophysiological mechanisms of various diseases. Among other biological fluids, saliva is attracting interest from researchers because its collection represents a noninvasive method to evaluate oxidative changes. Despite a relatively large number of studies reporting the association of oral diseases with changed oxidative stress markers in saliva, reliable data on salivary oxidative stress markers in healthy individuals are lacking.

It has been shown that blood oxidative stress markers can vary over time depending on season and location [27, 28]. However, attempts to compare markers from blood and saliva have not resulted in clear conclusions. Several studies have shown that salivary biomarkers correlate well with serum levels [29]. Yet other studies have noted that the values of antioxidants in saliva do not correspond to blood values, demonstrating their specificity in relation to various oral pathologies [30, 31].

Due to the paucity of research on salivary oxidative stress levels, our aim was to evaluate diurnal variations and day-to-day fluctuations of salivary oxidative stress markers in healthy adult individuals. An understanding of the day-to-day variability of these markers could serve as standardized reference data, allowing researchers to better interpret future results and make future comparisons.

It should be noted that in our study, salivary biomarker levels were reported as normalized for saliva total protein concentration. We corrected for total protein concentration in order to overcome variability associated with sample dilution. The technical repeatability of the used tests showed satisfactory analytical precision to be used clinically.

Studies that analyzed the effect of gender on oxidative stress markers mostly report significant gender-related differences. In the study by Lettrichová et al. [25], all measured salivary markers of oxidative stress and antioxidant status in healthy individuals were lower in women than in men. The authors suggested smaller size of salivary glands and a lower unstimulated salivary flow as a possible explanation for the observed differences. Sculley and Langley-Evans [32] also reported significantly lower salivary total antioxidant status in women compared to men, regardless of periodontal health. Similar gender-related differences were also shown in the study assessing oxidative stress markers in blood [33]. These are usually attributed to differences in sex hormones, especially to the antioxidative properties of estradiol. However, other studies, including the results of the present study, suggest that gender has no effect on the levels of oxidative stress biomarkers. In a study by Ahmadi-Motamayel et al. [34], a comparison of male and female subjects showed no statistically significant differences in their TAC and MDA levels in both salivary and serum samples. Behuliak et al. [26] did not find gender-related difference in the dynamics of salivary TBARS levels. Additionally, in a study by Kamodyová et al. [24], no significant differences in salivary levels of AOPP, TBARS, AGEs, FRAP, and TAC were observed between men and women. Factors underlying conflicting results concerning the influence of gender on the levels of oxidative stress markers are yet to be elucidated.

When evaluating intraindividual differences, salivary MDA showed the largest variability. This is in accordance with the results of the study by Behuliak et al. [26] who reported high intraindividual variability for salivary TBARS in both genders with CVs of more than 60%. Furthermore, in 40% of the subjects, CVs of more than 35% were observed for 8-OHdG, which also signals poor intraindividual variability. For SOD, TAC, GPX, and UA, good-to-moderate interday CVs were observed in more than 75% of the subjects. Lettrichová et al. [25] reported high intraindividual variability of salivary OS markers, from 20% for TAC to 30% for AGEs and FRAP and 45% for AOPP. The results of both studies were based on 30 consecutive days of sampling [25, 26]. Over such a long period, several other factors, such as dietary changes or respiratory tract infections, could influence the increase of variability. Based on the high variability in the salivary concentrations of MDA and 8-OHdG obtained in our short-term study, we conclude that levels of those biomarkers cannot be reliably ascertained based on single measurements. Instead, multiple measurements, again repeated over several days, would provide more reliable and precise information.

For all examined salivary oxidative stress markers, no significant day-to-day variations were detected. However, a significant diurnal effect for GPX, TAC, and MDA, with significantly lower values in the morning than in the afternoon, was observed. In view of this effect on these markers, consideration should be given to the time of the day samples are collected. No such effect was observed for salivary SOD, UA, or 8-OHdG. Circadian variations in salivary concentrations of OS markers were examined by Kamodyová et al. [24], who noted significantly higher levels of salivary AGEs in the morning and salivary FRAP in the afternoon but found that salivary TAC did not differ significantly during the day. Maximal concentrations in salivary TAC during early morning hours (6 AM), observed by Borisenkov et al. [35]., have been explained by a possible influence of melatonin on increasing morning TAC, because correlation between serum TAC and melatonin has been described as strong, direct, and significant [36–38]. However, a noticeable lag was acknowledged between the time of maximum production of melatonin and maximum TAC of saliva. Contrary to the previous results, our study demonstrated lower salivary TAC measured in the morning samples compared to that taken in the afternoon hours. It could be presumed that, similarly with the results of the studies demonstrating day-night differences in the levels of serum TAC [37, 38], basal salivary TAC in healthy adults with good oral hygiene could be low in the morning hours. Comprehensive studies assessing the correlations between serum and salivary oxidative stress markers are missing, but there are reports suggesting a significant relationship between selected markers in serum and saliva [39, 40]. Somewhat higher TAC, as well as GPX and MDA concentrations in our afternoon saliva samples, could be explained by external factors that may influence the levels of markers of oxidative stress and antioxidant status during the day. These include dietary habits [41], physical activity [42], usage of mobile phones [43], watching television [44], and psychosocial stress [45]. Diurnal variations in salivary concentrations of other OS markers have not been examined previously, but some studies have reported a significant circadian rhythm of blood SOD, GPX, and MDA in healthy volunteers; similar to our results, mean GPX and MDA activity were observed to be at maximum at 6 PM [46].

Our study demonstrated that reliability across the three days varied depending on the marker. For GPX, reliability was higher if sampling was performed in the morning, whereas afternoon sampling showed greater reliability for MDA and TAC. For other OS markers, similar reliability was observed for morning and afternoon testing. Between-day variances over the three test days were less than 10% for GPX, SOD, UA, and TAC, but we observed relatively high between-day variance (CV > 20%) for salivary MDA. Wide discrepancies in salivary MDA concentrations of healthy subjects have been recorded previously, with MDA values varying from 27 to 680 to 900 nmol/L [47–49], which suggests the need for validation of MDA measurements. These wide variations might be due to the method of saliva collection, the storage of samples prior to analysis, or the analytical method used [4]. In our study, even after normalizing MDA for saliva total protein concentration, wide variations were observed. We determined MDA by using an ELISA assay that measures the quantity of MDA adducts, given MDA's capability to bind to proteins and form stable adducts. Since MDA readily combines with different functional groups on various molecules, it is useful to measure MDA protein adducts because its quantity reflects the amount of MDA involved in the formation of advanced lipid peroxidation end products. However, the amount of MDA adduct in the used standards was predetermined by a TBARS assay kit, as declared by the manufacturer, allowing our results to be compared with other studies analyzing MDA. Due to the large variability in salivary MDA levels, we suggest multiple measurements of MDA over several days in order to obtain more dependable data.

All OS markers showed wide between-subject variability. The group CV% ranged from 30.08% to 75% for morning and 30.28% to 85.70% for afternoon measurements. High between-subject variability of salivary oxidative stress markers was also reported in other studies [25, 26]. Therefore, when interpreting the results of oxidative stress marker comparison between orofacial pain patients and healthy controls, this high between-subject variability should be taken into account.

The present pilot study is observational, describing the variability of oxidative stress markers in healthy individuals. The biggest limitation of the study, which reduces the strength of our conclusions, is its small sample size. Studies that involve larger populations are needed to address the issues; this will be the focus of our future investigations. Another limitation that could have affected our results is the single measurement of the parameters. However, all measurements were performed in an accredited laboratory, by a single analyst who applied all principles of good laboratory practice.

## 5. Conclusion

Due to large intraindividual variability in the salivary concentrations of MDA and 8-OHdG, we conclude that the levels of these markers cannot be reliably determined based on single measurements and that multiple measurements taken over several days would generate information that is more accurate and reliable. Salivary SOD, TAC, GPX, and UA proved to be stable across the three days of measurement; therefore, single sampling might adequately reflect the level of these markers. These findings are of utmost importance for objectively assessing the results that have been and will be obtained by studies investigating salivary oxidative stress markers in various conditions. Without adequate consideration of these variations and reliability results, any study on this topic will lack proper interpretation.

## Figures and Tables

**Table 1:** Within-subject test-retest reliability data.

	Morning						Afternoon					
	Sampling day mean values			Mean (SD)	ICC	Between-day CV (%)	Sampling day mean values			Mean (SD)	ICC	Between-day CV (%)
	1	2	3		*r* (95% CI)		1	2	3		*r* (95% CI)	
GPX (U/g)	112.3	119.1	112.9	114.8 (3.8)	0.86 (0.66–0.95)	3.28	154.4	143.7	156	151.4 (6.7)	0.61 (0.06–0.85)	4.40
SOD (U/g)	2801	2725.5	2959.6	2828.7 (119.5)	0.76 (0.39–0.91)	4.22	2636.3	2399.6	2793.6	2609.9 (198.3)	0.77 (0.46–0.91)	7.60
TAC (mmol/g)	1.6	1.7	1.6	1.6 (0.1)	0.76 (0.43–0.91)	4.06	2.2	2	2.2	2.1 (0.1)	0.81 (0.55–0.93)	6.83
UA (umol/g)	323.1	346.1	350.9	340 (14.9)	0.72 (0.32–0.89)	4.38	422.8	414	440.6	425.8 (13.6)	0.51 (0.15–0.82)	3.19
8-OHdG (ug/g)^∗^	2.6	2.3	2.3	2.4 (0.2)	0.82 (0.57–0.93)	6.68	2.2	2.4	1.8	2.1 (0.3)	0.83 (0.59–0.94)	14.5
MDA (nmol/g)^∗^	665.9	534.1	393.1	531 (136.4)	0.34 (−0.6–0.76)	25.69	1523.9	1142.8	1057.2	1241.3 (248.5)	0.86 (0.66–0.95)	20.01

GPX: glutathione peroxidase; SOD: superoxide dismutase; TAC: total antioxidant capacity; UA: uric acid; 8-OHdG: 8-hydroxydeoxyguanosine; MDA: malondialdehyde; SD: standard deviation; ICC: intraclass correlation coefficient; CI: confidence interval; CV: coefficient of variation. ^∗^Log transformation required before ICC calculation, due to nonnormal distribution.

**Table 2:** Between-subject means, standard deviations (SD), and group coefficient of variation (CV).

	Morning		Afternoon	
	Mean (SD)	Group CV (%)	Mean (SD)	Group CV (%)
GPX (U/g)	114.8 (57)	49.66	151.4 (61)	40.32
SOD (U/g)	2828.7 (872.2)	30.83	2609.9 (1494)	57.24
TAC (mmol/g)	1.6 (0.5)	30.08	2.1 (0.7)	33.04
UA (umol/g)	340 (138.4)	40.71	425.8 (128.9)	30.28
8-OHdG (ug/g)	2.4 (1.8)	75.00	2.1 (1.8)	85.7
MDA (nmol/g)	531 (314)	59.13	1241.3 (1026)	82.65

GPX: glutathione peroxidase; SOD: superoxide dismutase; TAC: total antioxidant capacity; UA: uric acid; 8-OHdG: 8-hydroxydeoxyguanosine; MDA: malondialdehyde.

## Additional Points

*Availability of Data and Material*. The datasets generated and analyzed during the current study are not publicly available, because this is a pilot study and further study will include orofacial pain patients who are still being recruited. However, all data from this study is available from the corresponding author on reasonable request.
